# From aging worms to the influence of the microbiota: an interview with David Weinkove

**DOI:** 10.1186/1741-7007-11-94

**Published:** 2013-08-29

**Authors:** David Weinkove

**Affiliations:** 1 School of Biological and Biomedical Sciences, Durham University, South Road, Durham DH1 3LE, UK

## 

David Weinkove did his undergraduate degree at Cambridge, then a PhD at University College London with Mike Waterfield and Sally Leevers. His first postdoctoral position was with Ronald Plasterk at the Hubrecht Laboratory in the Netherlands, followed by another with Nullin Divecha at the Netherlands Cancer Institute. He then went to David Gems’ lab at University College London, where he worked on the study of aging in *Caenorhabditis elegans*, before moving to Durham University in 2008.

We spoke to him about the paper he published with David Gems in *BMC Biology* [1] when an apparently straightforward experiment on *C. elegans* longevity took a surprising turn that refocused Weinkove on the *Escherichia coli* that the worms feed on and the influence of the microbiota on health and longevity.

**Figure:**
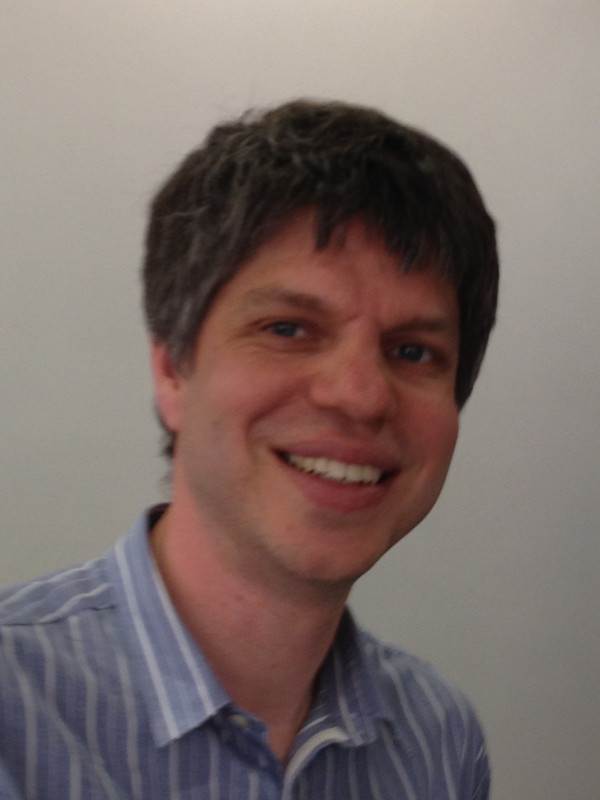
David Weinkove.

## The research that you’ve published with us in *BMC Biology* started off with a screen for genes that affect longevity in the classic longevity animal, the *C. elegans* nematode worm, but unexpectedly led you into insights into the effect of the microbiome. How did that happen?

I was in David Gems’ lab, where we were looking for genes that influence life span. A very nice thing that you can do with *C. elegans* is to knock out genes simply by feeding them *E. coli* that have a plasmid in them that contains RNAi for the worm gene you’re interested in. *E. coli* is the standard diet for *C. elegans* in the lab - growing *C. elegans* on lab strains of *E. coli* is just something that Sydney Brenner decided to do when he started working on *C. elegans* in the late sixties. He just did it because it was convenient, and 40 years later, *C. elegans* biologists all over the world do exactly the same thing with exactly the same strains. So I was feeding worms *E. coli* that was knocking out some genes that we thought might be involved in lifespan.

And with one of these *E. coli* strains, which we expected to make the worms shorter-lived, the worms actually lived dramatically longer. In biology, you don’t ignore results like that. If you see a big effect, you go after it. For quite a long time I thought this *E. coli* was knocking out a particular *C. elegans* gene. And then one day, I just noticed that there was a very subtle difference in color between the strain I was using and the control strain. I showed it to a PhD student, and he couldn’t see the difference. But because I had been looking at this *E. coli* strain for such a long time, I could see there was a slight difference. So I thought well maybe this is not to do with the worm gene, maybe it’s something that has happened to this *E. coli* strain. So I got rid of the plasmid DNA that was knocking out the gene from *C. elegans* and found that this particular *E. coli* strain still made the worms live longer. It was nothing to do with knocking out the worm gene - something had happened to the *E. coli* strain. That made us think there was some sort of spontaneous mutation that occurred in this *E. coli* strain and made the worms live longer. We found what it was when we found that these *E. coli* grew poorly on medium lacking glycine, and it turned out that the gene that was disrupted was a metabolic gene involved in making aromatic compounds. And by adding back different compounds, we pinned it down to PABA, which is the precursor of folates, a broad term which includes folic acid. Folates are essential for life.

## And in fact are added to our diet in various ways

Yes, in the US they supplement flour with it. In the UK we have it in cereals and margarine. It’s important. The supplementation is done to prevent neural tube defects, but it’s actually very important generally for growth. The funny thing is animals don’t make it. So we need to get it either from our diet or from our microbes. Microbes in the gut make a lot of folic acid, and that would be the main source of folates for *C. elegans*.

So, I moved to set up my own lab in Durham. We started using some inhibitors of folate synthesis to try and get the same effect as the *E. coli* mutant. The sulfonamide drugs inhibit folate synthesis in microbes, and my PhD student found that the more sulfonamide you add, the longer the worms live. You can do this just by using a drug. That’s how we got to the discovery that we reported in our *BMC Biology* paper - it was just an accidental discovery that led to finding that inhibiting folate synthesis in the microbes makes the worms live longer.

## The obvious question is - if sulfonamide drugs do this to worms, what do they do in mammals?

Well that was something interesting that we found out when we went to the Patent Office, because Durham University tries to make sure that we protect our intellectual property. The Patent Office found a paper published in 1958, and written in German, from Bayer, which is the German company that originally discovered sulfonamides. They had been trying to see if the drugs were useful in treating cancer, and they made the incidental observation that mice and rats and even dogs that were given sulfonamides lived longer [2].

Now that was a little bit anecdotal and it needed to be followed up, but it was quite an interesting observation. And much more recently, Jeffrey Gordon’s lab, where they are very interested in the microbiome, looked at changes in microbial genes across all microbes in the human gut and how they change at different ages [3]. The gene class that they picked up - I think it was the strongest one - and that they put in their paper, was to do with folate synthesis. This is really more to do with development than with aging. Before two years old, microbial folate synthesis genes are highly represented. After two, the folate synthesis genes go down and you get folate salvage genes. What we think is that a lot of microbes are making a lot of folate and that’s why some other microbes need to salvage it - maybe this indicates that too much folate is bad for you.

## When you say too much, too much for what?

Too much for growth. Folate works as an enzyme. It’s needed for biosynthesis - but it’s not actually consumed in the biosynthesis, it works as a coenzyme. You only need a very small amount of folate. The really fascinating thing was, if you treat a microbe with a sulfonamide you’d think you’d kill it off, but we don’t. We don’t see any effect on the growth of bacteria with the sulfonamides. When you measure the folate levels, they come right down, but there’s still enough folate for the bacteria to grow fine. They’re obviously making much more than they need in our system. Then there’s some other old data that show that quite a lot of microbes make and excrete folate. So in terms of what’s going on - now, microbes don’t do anything for no reason. There must be a reason for all this folate synthesis - we just don’t understand what it is. We think they’re making more than they need, but we haven’t worked out if that’s actually happening *in vivo*. We have to try and look at real microbes, microbes from the mammalian gut - is this really happening there?

## So should we all be concluding that too much folate is bad for us for reasons we don’t understand?

Well no, this is the interesting thing. More recently we’ve found that even though we treat the microbes with sulfonamides so they get less folate and there’s less folate in the worm, it’s not the decreased folate in the worm that’s making the worms live longer. We know this because we can supplement with folinic acid, which is a more natural supplement than the stuff that’s used in supplements for cereals and other foods. A small dose of folinic acid can directly restore the folates in the worm to high levels without affecting the gut bacteria, and that manipulation doesn’t affect the lifespan effect. So it could be that folates are good for you in your body, but having too much folate in your gut microbes might cause the gut microbes to do something that’s not very good for you.

## So does this mean we need to look at what people have called the holo-biome - not just the organism, but all the microorganisms associated with it - in order to understand the effects of any metabolic interference - is that the bottom line?

I think that’s important, yes. We’ve co-evolved with microbes. That’s the whole system - the microbes’ interaction with each other and with the host. Whether it’s a metabolic thing, I’m not sure. It seems that there is an effect of folates - something that’s going on in the microbes that are making too much folate - that has an effect on the host. And that raises an interesting point about the supplements. We were confused for a long time because when we gave folic acid, which is the traditional oxidized folic acid that you find in supplements, we found that we did reverse the lifespan effect. But then we found that what happens with that folic acid is that there’s a breakdown product that can be taken up and used by *E. coli*. Now that has some ramifications because when we’re taking our folic acid supplements, we’re going to be absorbing some of it, but some is going to be broken down and absorbed by our microbes. So although some microbes take up the folic acid directly, *E. coli* and many others take up the breakdown product. So maybe it’s better to use different sorts of supplements than folic acid.

## Are you suggesting that any sort of supplement or drugs that we might take, we need to understand what they’re doing to the gut microbiota before we can understand what they’re likely to do to us?

I think there’s a very high chance that any drug or supplement that we take will be metabolized by gut microbes, and will have an effect on us potentially through gut microbes.

## So what is your own lab pursuing now? Are you still looking at longevity or has this changed the focus of your research?

We are still looking at longevity. We still need to understand what it is about excess folate in the microbes that’s effectively limiting the lifespan, so that when we take it out the worms live longer and are healthier. As I said, this gene was found by accident. Maybe there are other genes out there. We’ve started doing screens for other genes in *E. coli* that might also affect lifespan. But the folic acid effect is a really robust thing. It has stood the test of a lot of experiments in our lab. We’re trying to see if this is applicable in mammalian systems, if it’s applicable in other forms of microbes - not just the lab strain that Sydney Brenner decided to use - but actually in microbes that have been implicated in disease or microbes straight out of the human gut.

## So it’s driven you up the phylogenetic tree to mammals and out from lab *E. coli* into the rest of the microbiome. That seems a very large step to have taken

It’s interesting because my background is working with *C. elegans* but now I’m working with *E. coli* a lot more. But at the same time, as you say, it has more relevance potentially to human health than perhaps some of the more direct *C. elegans* work.

So, for example, treating microbes with antibiotics is not a good idea. You don’t want to kill off your microbes, you need to keep the balance of your microbiota. So what’s interesting about our experimental intervention is that it doesn’t seem to affect the growth of the microbes. So it gives us hope that it might be useful. All the drugs developed against microbes have been about killing the microbe. Actually maybe the new class of drug is about modulating the metabolism of the microbes without killing them and that’s the way to go. That’s what we’re trying to think about doing. Because you can’t actually give someone a sulfonamide, like SMX, long-term. It accumulates in the thyroid and causes problems. What we want is a drug that doesn’t go into the body, stays in the gut, affects the microbes, doesn’t really harm the microbes, just tweaks their metabolism. That’s what you want I think. Otherwise you get resistance and loss of diversity. It might be that natural foods tweak microbial metabolism already. Green tea or something like that. One thing that the microbial field is going away from - it’s not so much about which species of bacteria you have, but which metabolic pathways are active. That gives us hope that just working with this single strain of a single microbe might still be useful because you’re finding pathways that are also in other microbes. So the focus is moving towards metabolism in the microbes, rather than just what one species does and what another species does.

## So really I was wrong when I said you’re moving up the phylogenetic tree - you’re really moving down, from *C. elegans* to *E. coli*

Well, yes. You know, we only know what about half the genes in *E. coli* are doing - 1,700 out of 4,000 are of unknown function, probably to do with interactions with the environment. Worms are really great for their convenience and ease of breeding, and they’re inexpensive to maintain - a great austerity organism. But now I see them more as a biosensor for what *E. coli* is doing.

## References

[B1] Virk B Correia G Dixon DP Feyst I Jia J Oberleitner N Briggs Z Hodge E Edwards R Ward J Gems D Weinkove D Excessive folate synthesis limits lifespan in the C. elegans: E. coli aging model BMC Biol 2012 10 67 10.1186/1741-7007-10-67 22849329 PMC3583181

[B2] Hackmann C Observations on influenceability of age phenomena in experimental animals by peroral administration of combinations of 2-(p-aminobenzolsulfonamide)-pyrimidin Munch Med Wochenschr 1958 100 1814 1817 13622503

[B3] Yatsunenko T Rey FE Manary MJ Trehan I Dominguez-Bello MG Contreras M Magris M Hidalgo G Baldassano RN Anokhin AP Heath AC Warner B Reeder J Kuczynski J Caporaso JG Lozupone CA Lauber C Clemente JC Knights D Knight R Gordon JI Human gut microbiome viewed across age and geography Nature 2012 486 222 227 22699611 10.1038/nature11053 PMC3376388

